# An all-dielectric metasurface as a broadband optical frequency mixer

**DOI:** 10.1038/s41467-018-04944-9

**Published:** 2018-06-28

**Authors:** Sheng Liu, Polina P. Vabishchevich, Aleksandr Vaskin, John L. Reno, Gordon A. Keeler, Michael B. Sinclair, Isabelle Staude, Igal Brener

**Affiliations:** 1 Sandia National Laboratories, Albuquerque, NM 87185 USA; 2 Institute of Applied Physics, Abbe Center of Photonics, Friedrich Schiller University Jena, Albert-Einstein-Str. 15, 07745 Jena, Germany; 3 Center for Integrated Nanotechnologies, Sandia National Laboratories, Albuquerque, NM 87185 USA

## Abstract

A frequency mixer is a nonlinear device that combines electromagnetic waves to create waves at new frequencies. Mixers are ubiquitous components in modern radio-frequency technology and microwave signal processing. The development of versatile frequency mixers for optical frequencies remains challenging: such devices generally rely on weak nonlinear optical processes and, thus, must satisfy phase-matching conditions. Here we utilize a GaAs-based dielectric metasurface to demonstrate an optical frequency mixer that concurrently generates eleven new frequencies spanning the ultraviolet to near-infrared. The even and odd order nonlinearities of GaAs enable our observation of second-harmonic, third-harmonic, and fourth-harmonic generation, sum-frequency generation, two-photon absorption-induced photoluminescence, four-wave mixing and six-wave mixing. The simultaneous occurrence of these seven nonlinear processes is assisted by the combined effects of strong intrinsic material nonlinearities, enhanced electromagnetic fields, and relaxed phase-matching requirements. Such ultracompact optical mixers may enable a plethora of applications in biology, chemistry, sensing, communications, and quantum optics.

## Abstract (web summary)

Frequency mixers are hard to achieve at optical frequencies because it is difficult to meet different phase-matching conditions. Here, the authors show that GaAs metasurfaces can mix laser beams to generate eleven new wavelengths through different nonlinear optical processes occurring simultaneously.

## Introduction

Mixers are devices that convert electromagnetic wave frequencies and are indispensable in signal processing. For example, radio-frequency mixers have been widely employed in modern communications and navigation systems as modulators, phase detectors, frequency synthesizers, heterodyne receivers, etc^1^. Frequency mixers are also in great demand at optical frequencies, where nonlinear crystals are used to generate new colors through nonlinear optical processes such as harmonic generation, sum-frequency and difference-frequency generation and high-order harmonic generation. These nonlinear optical processes have greatly broadened the accessible spectrum and are ubiquitous in applications ranging from cutting-edge science and technology to our daily life (such as green color laser pointers). Recent applications of nonlinear optical mixing include attosecond pulse generation^2^, supercontinuum generation^3^, optical frequency comb generation^4^, material characterization^5^, and quantum optics^6^. Until now, these applications have relied on bulk nonlinear crystals whose dimensions are much larger than the operating wavelengths and, to achieve efficient frequency conversion, the fundamental and newly generated frequencies need to travel in-phase (i.e., be phase matched) inside the nonlinear medium^7^. Consequently, dispersive isotropic materials such as GaAs, although possessing large nonlinear coefficients, cannot be used. Instead, birefringent materials with much smaller nonlinear susceptibilities such as lithium niobate and barium borate are widely employed. However, due to dispersion, phase matching can only be achieved for one nonlinear process within a narrow bandwidth, and wavelength tuning is achieved by varying incident angles, or using different crystals. Quasi-phase matching^8–11^ can be used to exploit the large nonlinearities of isotropic materials, but its utility is limited by narrow bandwidth operation, with spectral tuning being achieved by angular rotation, temperature tuning, or electric field bias. Therefore, a device that enables multiple frequency mixing processes across a wide spectral range can be a powerful and versatile platform—however such a device has not been realized using conventional nonlinear optics.

The emergence of nanoresonators, metamaterials, and metasurfaces has revolutionized our perception of nonlinear optical processes^12^. In contrast to bulk nonlinear optical crystals, subwavelength resonant cavities^13–16^, nanoparticles and nanoantennas^17,18^ greatly enhance electromagnetic fields in tight volumes^18,19^ and relax phase-matching conditions. This allows the simultaneous occurrence of various nonlinear processes^20–22^. More recently, semiconductor dielectric metamaterials, operating below the bandgap, have attracted intense attention due to their low material losses at optical frequencies^23,24^, as well as their resonant interaction with both the electric and magnetic fields^25^. In particular, odd order nonlinear optical processes including two-photon absorption^26^, third-harmonic generation (THG)^27^, and four-wave mixing (FWM)^28^ were studied in dielectric metasurfaces made from centrosymmetric semiconductors such as silicon and germanium. On the other hand, even order nonlinear effects such as second-harmonic generation (SHG)^29,30^ and sum-frequency generation (SFG)^22^ were not reported until the realization of III–V semiconductor nanoresonators or metasurfaces^23^, see Supplementary Note 1. However, simultaneous frequency conversion including both even and odd nonlinearity has not been observed using dielectric metamaterials.

Here, we demonstrate an optical metamixer—a GaAs-based dielectric metasurface that enables a variety of simultaneous nonlinear optical processes across a broad spectral range. Specifically, seven different nonlinear processes (second-harmonic, third-harmonic, and fourth-harmonic generation, sum-frequency generation, two-photon absorption-induced photoluminescence, four-wave mixing, and six-wave mixing (SWM)) simultaneously give rise to eleven new frequencies that span the ultraviolet to NIR spectral range. Our multifunctional metamixer exploits the combined attributes of resonantly enhanced electromagnetic fields at the metasurface resonant frequencies, large even-order and odd-order optical nonlinearities of non-centrosymmetric GaAs, and significantly relaxed phase-matching conditions due to the subwavelength dimensions of the metasurface.

## Results

### Samples

**Fig. 1:**
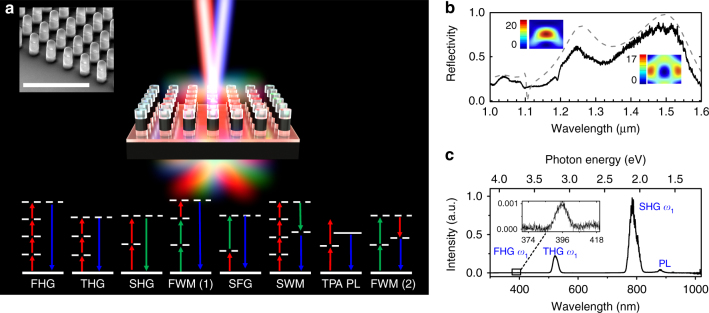
Generation of new frequencies with the GaAs metasurface mixer. **a** Schematic of an optical metamixer consisting of a square array of subwavelength GaAs dielectric resonators. Two femtosecond near-IR pulses pump the metamixer and a variety of new frequencies are simultaneously generated. Inset: a 60° side-view scanning electron microscope image of the GaAs metamixer. The scale bar corresponds to 3 μm. Bottom inset: schematic energy diagrams of the seven nonlinear optical processes that occur simultaneously in our metamixer: second-hamonic generation (SHG), third-harmonic generation (THG), fourth-harmonic generation (FHG), sum-frequency generation (SFG), two-photon absorption-induced photoluminescence (TPA PL), four-wave mixing (FWM) and six-wave mixing (SWM). **b** Measured (solid line) and numerically simulated (dashed line) reflectance spectra of the metasurface with two cross-section local electric field distributions at the wavelengths of 1.246 μm and 1.535 μm, which correspond to the maximal electromagnetic field enhancements inside the GaAs nanodisk. **c** Spectra of second-, third- and fourth-harmonics when pump pulses of *λ*_1_ ~ 1.57 μm are used to excite the GaAs metamixer. Inset is the zoom-in of the fourth harmonic generation

Figure 1a shows a schematic of nonlinear frequency generation by a GaAs metasurface pumped by two laser beams. The left inset of Fig. 1a shows a 60° side-view scanning electron microscope (SEM) image of a typical GaAs metasurface used in these measurements. The metasurface consists of a periodic square array of nanocylinders with a diameter of ~400 nm. The period of the structure was chosen to avoid coupling between the nanocylinders, as well as to have the highest filling factor. Each nanocylinder consists of three layers: the top SiO_x_ etch mask (~300 nm), the middle ~450 nm thick GaAs nanodisk that confines the electromagnetic field and the bottom low refractive index (Al_x_Ga_1−x_)_2_O_3_ layer (~400 nm) for isolating the GaAs nanocylinder from the high index GaAs substrate. Note that we used a (100)-oriented GaAs wafer for the sample fabrication; see Methods for further details. The resonantly enhanced frequency mixing is achieved by exciting the lowest order magnetic dipole (MD) and electric dipole (ED) Mie resonances of the GaAs nanocylinder^23,30–32^ simultaneously. The measured reflectivity spectrum (Fig. 1b) exhibits maxima at *λ*_ED_ ~ 1.25 μm and *λ*_MD_ ~ 1.5 μm which correspond to the excitation of the ED and MD resonances. This was confirmed by performing multipolar decomposition of the scattering fields, as well as by the simulated electric field profiles (shown in the insets) for the two wavelengths that correspond to maximum electromagnetic field enhancement inside the nanoresonators (1.246 μm and 1.535 μm). Note that these wavelengths occur in the vicinity of the reflectivity maxima, but the wavelength of maximum field enhancement of the MD resonance is slightly red-shifted.

### Harmonic generation in one-beam experiments

First, we study harmonic generation by the GaAs metasurface when pumped by a single near-IR femtosecond beam with a wavelength near the MD resonance (*λ*_1_ ~ 1.57 μm) using an average power of ~4.5 μW. We used a ×20 near-IR objective with a numerical aperture NA = 0.4 to both focus the pump pulses on the GaAs metasurface and collect the harmonic beams; for further details of experimental setup see Methods and Supplementary Note 3. Figure 1c shows the second-harmonic, third-harmonic and, fourth-harmonic (inset of Fig. 1c) generated by the metasurface at *λ*_SHG_ ~ 785 nm, *λ*_THG_ ~ 523 nm, *λ*_FHG_ ~ 393 nm, respectively. The efficiency of the SHG process is estimated to be 2.3 × 10^−6^; see Supplementary Note 4. The emission centered at *λ*_PL_ ~ 870 nm corresponds to GaAs photoluminescence (PL) arising from two-photon absorption (TPA) of the pump^33^. The observed harmonics lie above the GaAs bandgap energy and therefore suffer from significant material absorption. The conversion efficiency is much higher than the recently published record, which is high in SHG efficiency using mode-matched plasmonic nanoantennas^34^. Note, that in ref ^13,35^ even higher values of SHG efficiencies were reported due to the resonant nature of the optical nonlinearity created through a 3-level system using intersubband transitions in quantum wells. That design is not scalable beyond 3rd order nonlinearities, or to visible and near-IR wavelengths; nor can it be used to create simultaneous multiple-order nonlinearities.

### Frequency mixing in two-beam experiments

**Fig. 2:**
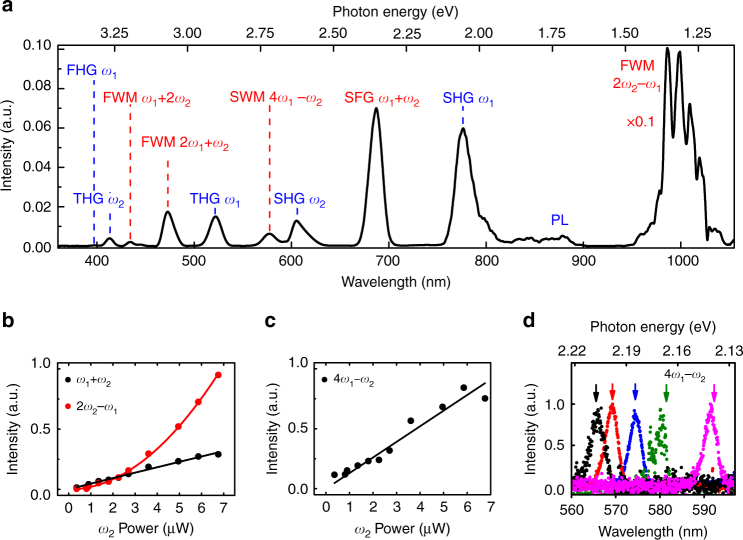
Frequency mixing in GaAs metasurface. **a** Spectrum exhibiting eleven nonlinearly generated peaks originating from seven different nonlinear processes when two optical beams at *λ*_2_ ~ 1.24 μm and *λ*_1_ ~ 1.57 μm are used to simultaneously pump the GaAs metasurface. Blue labels indicate harmonic generation processes and photoluminescence arising from two-photon absorption that each requires only one pump beam. Red labels indicate frequency mixings that involves both pump beams. **b**, **c** Dependence of the sum-frequency generation $(\omega _1 + \omega _2)$, four-wave mixing $(2\omega _2 - \omega _1)$, and six-wave mixing (4*ω*_1_−*ω*_2_) intensities on the power of the *ω*_2_ pump. Both the experimental data (dots) and theoretical fitting (black line for linear fitting and red curve for quadratic fitting) are shown. **d** Five representative spectra showing the tuning of the normalized six-wave mixing $(4\omega _1 - \omega _2)$ signal when the pump wavelengths are spectrally tuned to *λ*_2_ ~ 1248.7 nm, *λ*_1_ ~ 1557.5 nm (black curve); *λ*_2_ ~ 1234.8 nm, *λ*_1_ ~ 1558.6 nm (red curve); *λ*_2_ ~ 1211.6 nm, *λ*_1_ ~ 1558.9 nm (blue curve); *λ*_2_~1234.9 nm, *λ*_1_ ~ 1581.2 nm (green curve); and *λ*_2_ ~ 1233.7 nm, *λ*_1_ ~ 1600.4 nm (magenta curve). The arrows denote the theoretically expected frequencies for the considered six-wave mixing process

Next, we introduce a second femtosecond pump beam spectrally tuned to *λ*_2_ ~ 1.24 μm to overlap the resonators’ ED mode. The collinearly propagating pump beams were focused at the same location on the sample with average powers of *P*_1_ ~ 3.6 μW and *P*_2_ ~ 5 μW, respectively. When the two pump beams are temporally coincident, we observe eleven spectral peaks, ranging from ~380 to ~1000 nm (Fig. 2a).

We categorize the generated signals into two groups. The first group, indicated by the blue labels, corresponds to harmonic generation processes and also PL arising from two-photon absorption. Each of the processes in this group relies on only a single pump beam. In contrast, the second group, indicated by red labels, corresponds to frequency mixing processes that require both pump pulses. The five frequency mixing signals include: sum-frequency generation ($\omega _1 + \omega _2$) at *λ*_SFG_ ~ 689 nm; three types of four-wave mixing ($2\omega _2 - \omega _1$, $2\omega _1 + \omega _2$, $2\omega _2 + \omega _1$) at ~1000 nm, ~472 nm, and ~434 nm, respectively; and six-wave mixing $\left( {4\omega _1 - \omega _2} \right)$ at *λ*_SWM_ ~ 577 nm. Note, that the amplitude of the FWM $(2\omega _2 - \omega _1)$ peak is at least ten times higher than any of the other nonlinear processes. This is attributed to the much lower absorption below the GaAs bandgap in contrast to the strong attenuation experienced by the other signals. Altogether, the measured spectra contain simultaneous contributions arising from seven nonlinear optical processes.

We verify the physical origin of the frequency mixing processes by measuring the output spectra for various pump wavelengths and for different pump powers. For example, the SFG output is identified due to its energy coinciding with the sum of the photon energies of the two pump beams $\omega _{\mathrm{{SFG}}} = \omega _1 + \omega _2$, as well as by the linear intensity dependence on one pump power when the other pump power is held constant (Fig. 2b black curve). In a similar manner, we performed power dependence measurements to verify the four-wave and six-wave mixing processes. The red curve in Fig. 2b shows the quadratic dependence of the FWM $(2\omega _2 - \omega _1)$ output on the $\omega _2$ pump power, and Fig. 2c shows a linear dependence of the SWM intensity on the power of the pump at $\omega _2$. Other power dependence curves are shown in Supplementary Note 5. To further confirm the SWM process, we spectrally tuned both pump wavelengths and observed excellent agreement between the measured and calculated SWM peak locations (Fig. 2d), see Supplementary Note 6. To verify that the observed nonlinear processes are enhanced by pumping at the dipolar Mie resonances, we performed similar frequency mixing measurements on both the unpatterned GaAs substrate and other GaAs metasurfaces with different diameter resonators, and as expected, significantly lower signal intensities were observed; refer to Supplementary Note 7.

### Pump–probe spectroscopy of newly generated frequencies

**Fig. 3:**
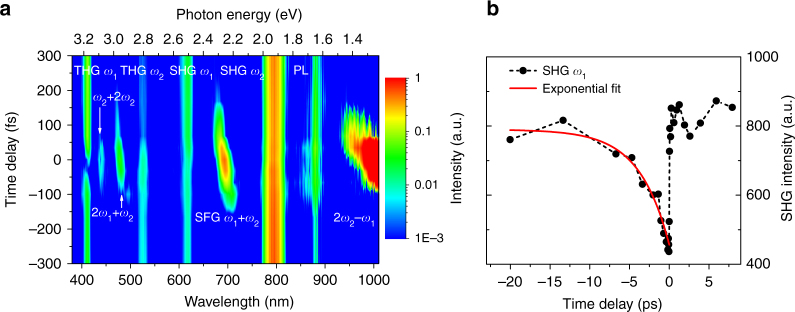
Temporal dynamics of nonlinear frequency mixing. **a** 2D contour image of the transient nonlinear signal (logarithmic scale) when the time delay between the two pump pulses is varied. The nonlinear signals that require only one of the pumps do not depend on the delay, while the mixing signals that rely on both pumps occur only when the two pumps overlap in time. **b** The quenching and the recovery of the $\omega _1$ SHG intensity due to the arrival of the second pump $\omega _2$ at the metamixer. The black dots are experimentally measured SHG intensities and the red curve is a single component exponential fit with time constant of ~3.7 ps

To investigate the temporal dynamics of the nonlinear generation processes, we measured the signal intensities while varying the optical delay between the two pump pulses. Figure 3a shows a 2D contour image of the transient nonlinear conversion. As expected, the harmonic generation signals and PL arising from two-photon absorption, which each requires only one of the pumps, are observed regardless of the optical delay. In contrast, the frequency mixing signals such as SFG, FWM, and SWM appear only when the two pump pulses temporally overlap at the metasurface. Note that we also observe time-dependent spectral shifts of the SFG and FWM signals. This is likely due to the chirp of the pump pulse: for example the negative chirp of $\omega _2$ causes the higher frequencies to arrive at the metasurface earlier than the lower frequencies; see Supplementary Note 8. More interestingly, we also observe significant changes of either pump’s harmonic generation intensities when the other pump illuminates the metasurface. For example, Fig. 3b shows the SHG dynamics of $\omega _1$, where the intensity decreases dramatically at zero delay and then recovers with a time constant of a few picoseconds (the red fitting curve has a single exponential component of 3.7 ps). While the details of the SHG quenching require further exploration and are beyond the scope of this paper, it is likely attributed to the spectral shift and broadening of the Mie resonance that in turn causes a reduction of the field enhancement inside the resonators. As we reported before^32^, resonance tailoring can be realized by refractive index modulation through free-carrier generation via one-photon absorption. In the current work, a lower energy but intense pump beam can efficiently generate free-carriers through TPA process; this is further supported by the observation of photoluminescence through TPA. The fast recovery of the SHG intensity (~3.7 ps) is due to the relaxation of the free-carriers through both nonradiative recombination at surface states^36,37^ and higher order processes such as Auger recombination^38^. Note that a longer recovery time constant such as thermal cooling could also exist^39^ but cannot be measured reliably due to the long-term instability of our laser system.

## Discussion

The nonlinearly generated signals shown in Figs. 1–3 were measured when the far-field collection angle was optimized for the SFG intensity, and we observe significant increases of the intensities of other signals when the collection angles are optimized individually. This indicates a variety of far-field emission profiles for different nonlinear signals, which significantly limits the measured conversion efficiencies, especially considering the low NA objective used^40^. Moreover, the majority of the nonlinearly generated frequencies lie above the GaAs bandgap and thus experience strong absorption. The conversion efficiencies can be improved by using larger bandgap materials such as AlGaAs to reduce absorption loss, or by fabricating resonators with larger dimensions so that the Mie resonances occur at longer wavelengths.

Dielectric resonators are a powerful platform that provides microscopic control over the electric field intensity and polarization distributions inside the resonator^40^. Higher conversion efficiencies for frequency mixing processes can be expected by engineering the resonator shape to optimize the overlap of the modes excited at the pump frequencies^28,30,40^. Moreover, III–V metasurfaces can be designed to tailor the polarization and far-field profiles of the nonlinear emission^13,16^ beyond what is possible with a single resonant particle^40^. The use of lower pump power might be possible when using dielectric metasurfaces based on high quality factor resonators which exhibit much larger electromagnetic field enhancements^41,42^. Another strategy for nonlinear optimization is to choose between (100), (110), and (111) orientations of the GaAs crystal. For example a *χ*^(2)^ process relies on the nonlinear polarizability $P^{\mathrm{{NL}}} \propto \chi _{{{ijk}}}^{\left( 2 \right)}E_{{j}}^{\omega _1}\left( {x,y,z} \right)E_{{k}}^{\omega _2}(x,y,z)$ where $\chi _{{{ijk}}}^{\left( 2 \right)}$ is the material’s intrinsic second-order nonlinear susceptibility, $E^{\omega _1}$ and $E^{\omega _2}$ are the fundamental pump fields; and *i*, *j*, and *k*, represent *x*, *y*, and *z* coordinates, respectively. Thus, for the electric field distributions and polarizations of a given resonator design, the crystal orientation can be chosen to maximize the nonlinear polarization within the resonator.

Our experimental demonstration of seven different nonlinear optical processes occurring simultaneously generated in GaAs metasurfaces could provide the opportunity for realizing ultracompact optical mixers for various applications. The observed even and odd high-order nonlinear processes might allow generation of high-order harmonics which is the foundation of attosecond pulse generation^43^. Moreover, we anticipate that these metasurfaces could be optimized for other nonlinear mixing processes such as difference frequency generation $\omega _{\mathrm{{DFG}}} = \omega _1 - \omega _2$. This would enable the production of femtosecond pulses covering the Mid-IR spectral range, where broadband laser-gain media and saturable absorbers do not exist.

## Methods

### Numerical calculations

To simulate the reflectance spectra of the GaAs metamixer, we used a finite difference time-domain simulator (Lumerical Inc.). The structural dimensions used in the simulation were: periodicity of 840 nm, SiO_x_ etch mask cap height of 300 nm and GaAs nanocylinder height of 400 nm and diameter of 400 nm. The half etched AlGaO bottom layer consists of two parts: the planar unetched oxide layer with a thickness of 200 nm and top oxide nanodisks with a height of 200 nm and diameter of 400 nm. The GaAs and SiO_x_ permittivity from the finite difference time-domain software were used in the simulation. The bottom AlGaO layer was considered a non-dispersive medium with refractive index of *n* = 1.6. The simulation was performed using an infinitely large (periodic boundary condition) metamixer structure excited by a normally incident plane wave.

To verify the excitation wavelengths of the ED and MD resonance, we performed full vector simulations in COMSOL. The elementary cell was excited by a normally incident plane wave. To perform the multipole decomposition of the scattered fields we implemented the method developed by Grahn et al.^44^.

### Sample fabrication

The metamixer was fabricated using standard electron-beam lithography and inductively coupled plasma (ICP) etch. The sample was grown using molecular beam epitaxy with a GaAs substrate at the bottom, a 400 nm thick Al_0.9_Ga_0.1_As in the middle and a 450 nm thick GaAs at the top (wafer VA0729). The semi-insulating GaAs substrate has a crystal orientation of (100), and therefore, the top GaAs layer has the same crystal structure. We started the process from spin coating a negative tone hydrogen silsesquioxane (HSQ Fox-16) electron beam resist. Circular disk patterns were written using 100 keV electron-beam lithography that converts the HSQ to SiO_x_. The unexposed resist was developed using tetramethylammonium hydroxide leaving ~300 nm tall SiO_x_ nanodisks as etch masks for GaAs. The shape of the SiO_x_ nanodisks was then transferred onto the GaAs and AlGaAs layers using a chlorine-based (both Cl_2_ and BCl_3_) ICP etch recipe. We controlled the etch duration so the AlGaAs layer was partially etched to half of the depth. Finally, the sample was placed in a tube furnace at ~420 °C for a selective wet oxidization process that converts the Al_0.85_Ga_0.15_As into its oxide (Al_x_Ga_1−x_)_2_O_3_ which has a low refractive index of *n* ~ 1.6. Note that the oxide has a larger lattice constant than AlGaAs, which causes the expansion of oxide thickness to be slightly larger than 400 nm.

### Reflectance spectroscopy measurement setup

The reflectance spectra of the sample were measured using a home-built white-light spectroscopy setup. The broadband light emanating from a halogen lamp was focused onto the sample using a ×20 Mitutoyo near-infrared objective. The numerical aperture (NA) of the incident light was minimized (NA < 0.1) by reducing the beam diameter before it entered the back aperture of the objective. The reflected light was collected by the same objective and detected by a liquid nitrogen cooled InGaAs camera that was connected to a near-IR spectrometer (Princeton Instruments monochromator/spectrograph 2500i). The reflectance was calculated by normalizing the reflected spectrum of a gold mirror measured under the same conditions. The experimental reflectance spectrum is shown in Fig. 1b.

### Nonlinear frequency mixing measurement setup

The two near-infrared femtosecond laser beams are generated using a twin-optical-parametric-amplifier that is pumped by an amplified Ti:sapphire laser with a 1 kHz repetition rate. The temporal duration (full-width at half maximum) of the optical pulses is 40–45 fs, which was measured using a frequency resolved optical gating technique. The wavelength of the two near-infrared beams can be tuned independently from 1.2 to 2.4 μm. The two beams are spatially combined by a dichroic beam combiner and then propagate collinearly afterwards. The temporal overlap between the pulses can be adjusted by a variable delay stage. Both beams are focused at the same spot on the surface of the sample by a ×20 near-infrared Mitutoyo objective with NA = 0.4. The focal spots are ~3 μm in diameter measured using the knife-edge technique. The average powers of the pump beams incident on the metamixer were varied between 0.3 to 7 μW (Fluence 3 mJ⋅cm^−2^ to 78 mJ⋅cm^−2^) for power dependent measurements.

The generated frequency mixing signals are collected by the same objective in a reflection geometry since most of the generated signals have photon energies above the GaAs bandgap and therefore would be completely absorbed in a transmission geometry. The resulting signal is then measured using a liquid nitrogen cooled silicon camera that is fitted to a spectrometer with visible gratings installed (Princeton Instruments monochromator/spectrograph 2300i). Note that only a portion of the generated signal could be collected by the low NA = 0.4 objective used in the setup.

### Relative spectral response calibration of the measurement setup

Due to the wavelength dependent response (such as transmission, reflection, diffraction) of the optical components (including the objective, dichroic beam combiner, lens, grating, silicon camera, etc.), the spectral efficiency of the frequency mixing setup needs to be calibrated accurately to allow calculation of the conversion efficiencies for the newly generated frequencies. First, a calibration lamp (Ocean Optics Calibration lamp HL-3plus-CAL) was placed at the focal spot of the objective to replace the sample. The rest of the setup was kept in the same condition as when the frequency mixing spectra were measured. Then, the lamp spectrum was measured using the visible spectrometer covering the wavelength range of the nonlinear signal. The relative spectral response of the setup was calculated by dividing the measured lamp spectrum by the calibrated lamp emission spectrum provided by the manufacturer. The frequency mixing spectra shown in the paper (for example Fig. 2a) were calculated considering the setup response and subsequently normalized to one. In Fig. 2a the measured data from 950 to 1080 nm were divided by a factor of 10 for a better visualization of the results. Due to the low silicon detector responsivity within this spectral region, the spectrum was also smoothed by averaging the neighboring data points to reduce noise.

### Data availability

The data that support the findings of this study are available from the corresponding author upon reasonable request.

## Electronic supplementary material

Supplementary Information
