# Supplementary material for: An all-dielectric metasurface as a broadband optical frequency mixer
Source: Nat Commun. 2018 Jun 28;9:2507. doi: 10.1038/s41467-018-04944-9 (PMC6023909; doi:10.1038/s41467-018-04944-9)
Supplement: Supplementary file 1 — Supplementary Information [file 41467_2018_4944_MOESM1_ESM.pdf]

An all-dielectric metasurface as a broadband optical frequency mixer

Liu et. al.

Supplementary Note 1

In this work, we measured frequency mixing processes from dielectric metasurfaces made from III-V semiconductors that include both even and odd nonlinearities. Specifically, we observed seven different nonlinear optical processes including second-, third-, fourth- and fifth-order nonlinearities that generate eleven new frequencies occurring simultaneously.

Supplementary Table 1 summarizes the nonlinear optical processes observed in our work compared with previously published results for both plasmonic and dielectric metasurfaces. Nonlinear processes up to third-order (third-harmonic generation (THG) and four-wave mixing (FWM)) were demonstrated before but with much lower conversion efficiencies. For dielectric metasurfaces, most works show only one nonlinear optical process, except a recently published paper, where both THG and FWM were observed in germanium nanodisks. However, germanium, like silicon, has a centrosymmetric crystal structure, so only odd order nonlinearities can be observed. Using non-centrosymmetric GaAs with high intrinsic nonlinearities, we not only present more nonlinear processes but also show both fourth-harmonic generation and six-wave mixing observed in metamaterials. Therefore, we envision GaAs dielectric metasurfaces as a suitable candidate for an optical frequency mixer for all orders. Supplementary Table 1 summarizes our results compared with previous work.

**This work (occurring simultaneously)**	**Plasmonic metamaterials**	**Dielectric metasurfaces**
Second-harmonic generation (2nd order)	Second-harmonic generation [1,2]	Second harmonic generation [3,4]
Sum-frequency generation (2nd order)	Third-harmonic generation [5]	Third harmonic generation [6]
Third-harmonic generation (3rd order)	Four-wave mixing [7]	Third harmonic generation and

Two-photon absorption induced Photoluminescence (3rd order, first time)<br>Three new frequencies generated by four-wave mixing: $(2\omega_2 - \omega_1, 2\omega_1 + \omega_2, 2\omega_2 + \omega_1)$ (3rd order, first time)<br>Fourth-harmonic generation (4th order, first time)<br>Six-wave mixing (5th order, first time)	2nd, 3rd harmonic and $(2\omega_2 - \omega_1)$ four-wave mixing [9]	Four-wave mixing [8]

Supplementary Table 1. Comparison of our work with previously reported studies of newly generated frequencies in nanostructures.

Supplementary Note 2

Simulated Reflectance Spectroscopy

The simulated reflectance spectrum of the GaAs metamixer is shown in Supplementary Figure 1a. The two reflectivity maxima at ~ 1.26 μm, and at ~ 1.496 μm, correspond to the resonant excitation of the magnetic dipole (MD) and electric dipole (ED) resonances, respectively. This was confirmed by performing calculations of multipolar electromagnetic field decompositions of the scattering spectra, shown in Supplementary Figure 1b for the 1st order dipole resonances. The simulated reflectance spectra and experimental measurements agree well. The minor differences likely originate from the slightly tilted sidewalls of the GaAs nanocylinders, as shown in the SEM image (left inset of Figure 1a). The red and blue arrows in Supplementary Figure 2a indicate the two wavelengths for maximum electromagnetic field

enhancement inside the GaAs nanoresonators, ~ 1.246 μm, and ~ 1.535 μm. Supplementary Figures 1c-d show the electric and magnetic field profiles at the two aforementioned wavelengths at a vertical plane located half way through the GaAs nanocylinder. The electric field inside the nanoresonator is 17× (MD) and 20× (ED) stronger than in free space. Note that the peaks of the enhancements, as well as the peaks in decomposition spectra are shifted from the reflectivity maxima, resulting in the red-shift of the λ_1 wavelength chosen for optical pumping of the MD resonance. This explains why our pump wavelength is slightly shifted from the measured reflectivity maximum.

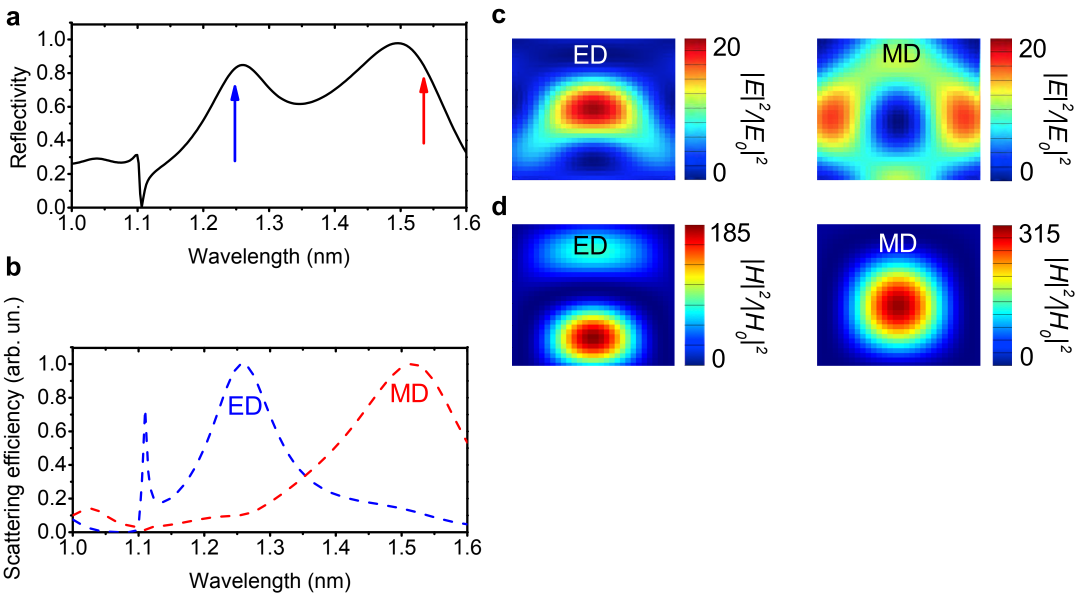

Supplementary Figure 1. Numerical calculations of the optical response of GaAs metasurface. **a** Simulated reflectivity spectrum of the GaAs metamixer. The red and blue arrows indicate the corresponding wavelengths of the maximal enhancement of electromagnetic fields at the magnetic dipole (MD) and electric dipole (ED) resonances, respectively. **b** Normalized simulated scattering efficiency spectra for MD and ED dipoles. Simulated (**c**) electric and (**d**) magnetic intensity profiles at the ED and MD resonances in the vertical plane located half way through the GaAs nanocylinder. The intensities shown by the scale bars are normalized to the incident field.

Supplementary Note 3

Nonlinear frequency mixing measurement setup

Supplementary Figure 2a shows the experimental setup for frequency mixing measurement. The spectra of the two near-infrared femtosecond laser beams and the reflectivity spectrum of the GaAs metasurface are shown in Supplementary Figure 1b.

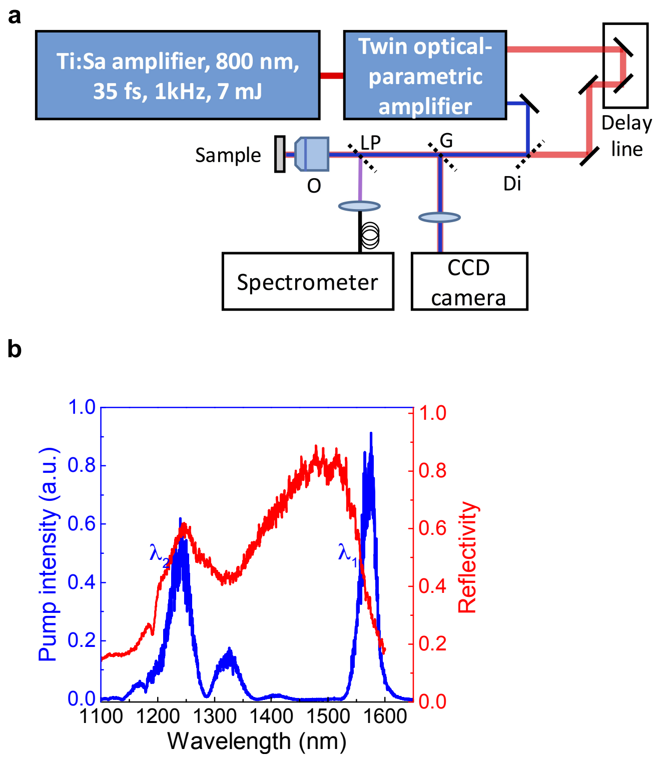

Supplementary Figure 2. Experimental setup for frequency mixing measurements. **a** Experimental setup for frequency mixing measurements using an amplified Ti:sapphire femtosecond laser system that enables independent spectral tuning of pump beams. Di: dichroic beam combiner, G: glass window, LP: 1064 nm long pass filter, O: near-IR objective. **b** The spectra of the two laser pump beams, λ_1 and the $\lambda_{2,}$ and reflectivity spectrum of the metamixer showing the magnetic dipole (MD) and electric dipole (ED) resonances.

Supplementary Note 4

Derivation of the conversion efficiencies for frequency mixing signals

To accurately measure the conversion efficiency of the frequency mixing signals, we needed to correlate the emitted power and the photon counts recorded using our spectrometer. Due to the low repetition rate (1 kHz) of the amplified Ti:sapphire laser system, the power of the nonlinear signal was too low to be measured using a Si-photodiode power meter. Therefore, we performed second-harmonic generation experiment using the same setup but pumped the metamixer with an 80 MHz repetition rate femtosecond laser. The pump laser (Toptica fiber laser) output has pulses with λ~1550 nm central wavelength which is also close to the MD of the metamixer. Despite the lower peak power, due to the much higher repetition rate, we were able to pump the sample with a relatively high average power of 10 mW (compared with a few micro watts using the amplified laser) and the generated second-harmonic reached a power level of dozens of nW, which is detectable by the power meter. Meanwhile, the photon counts of the second-harmonic were measured using the spectrometer. Therefore, a correlation between the power and photon counts was established.

By converting the photon counts of the frequency mixing signals to power, a conversion efficiency of ~2.6×10^{-6} was derived for the SHG process when only one pump beam was used to excite the metamixer. Note that this efficiency is likely limited by the collection efficiency of the low NA=0.4 objective. Through this measurement we were able to derive the conversion efficiency for any other frequency mixing processes since the relative conversion efficiency was calculated and explained above. For example, we estimate the conversion efficiencies for the sum-frequency generation, four-wave mixing ($2\omega_2 - \omega_1$), third-harmonic generation ($3\omega_2$) and fourth harmonic generation ($4\omega_2$) are ~3×10^{-6}, ~5×10^{-5}, ~6.4×10^{-7} and ~2.6×10^{-9}.

Supplementary Note 5

Verification of nonlinear optical processes by power dependent measurements

To verify the nature of the nonlinear optical processes we performed pump power dependent studies. We varied the pump power using a continuous variable neutral density filter. Supplementary Figure 3 shows the power dependent experimental results of several processes including second-harmonic generation, measured in one-beam experiment, two-photon absorption induced photoluminescence, sum-frequency generation, four-wave mixing, and six-wave mixing, measured with two-beams pumping the sample. The fitting functions used are based on the expected nonlinear power dependence. For example, we used a quadratic function for second-harmonic generation. Overall, the agreement is good.

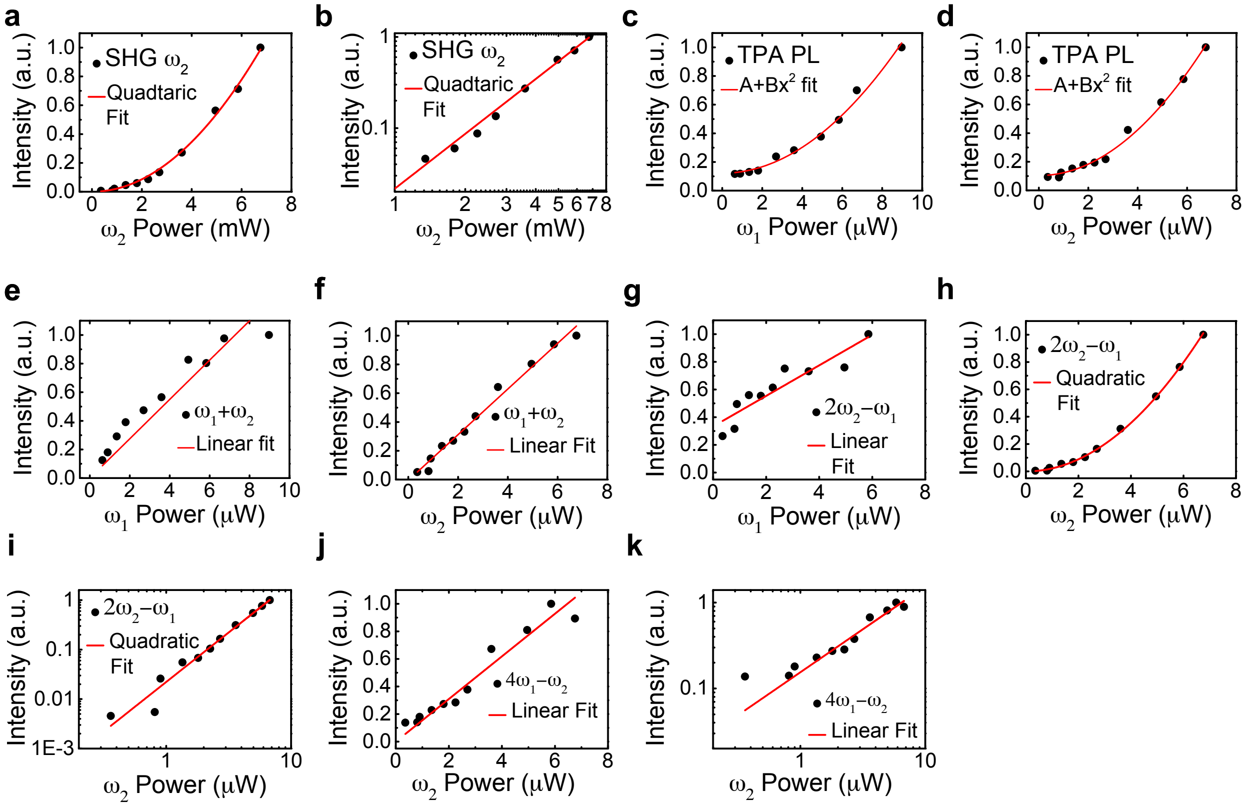

Supplementary Figure 3. Power-dependent measurements of generated nonlinear signals. Black dots are experimental data and the red curves are fitted using the expected nonlinear processes. The dependence of the second-harmonic generation (SHG) $\boldsymbol{\omega_2}$ intensity on the pump power shown in (a) linear and (b) logarithmic scales. c and d The dependence of the two-photon absorption induced photoluminescence (TPA PL) intensity on the power of either the first or the second pump beam, respectively. e and f The dependence of the sum-frequency generation intensity on the power of either the first or the second pump beam, respectively. g The dependence of four-wave mixing ($2\omega_2$- ω_1) on the power of the first pump beam. h and i The dependence of four-wave mixing ($2\omega_2$- ω_1) on the power of the second beam in linear and logarithmic scales. j and k The dependence of six-wave mixing ($4\omega_1$- ω_2) on the power of the first beam in linear and logarithmic scales.

Supplementary Note 6

Confirmation of the six-wave mixing process by spectral tuning

To confirm the nonlinear process of six-wave mixing (SWM), we performed both the pump power dependent measurement as well as tuning of the SWM spectral position by varying the wavelengths of the two pump beams. Due to the low conversion efficiency of the SWM process, spectral tuning renders higher accuracy than power dependent measurement. To isolate the SWM signal from other processes, we used two narrow bandwidth pass filters (full width at half maximum of ~8.5 nm) to reduce the spectral width of the two pump beams significantly. Supplementary Figure 4a shows the spectra of the two pumps, which is much narrower than the original bandwidth shown in Supplementary Figure 2b. A representative frequency mixing spectrum is shown in Supplementary Figure 4b. Figure 2d of the main paper shows the isolated SWM signals compared to the calculated positions. The summary of the five SWM signals are listed in Supplementary Table 2 where experimental results are compared with calculated spectral positions. The pump wavelengths were identified using the second-harmonic generation signal ($\lambda_{pump} = 2\times\lambda_{SHG}$). Excellent agreement between calculated and experimental results was achieved.

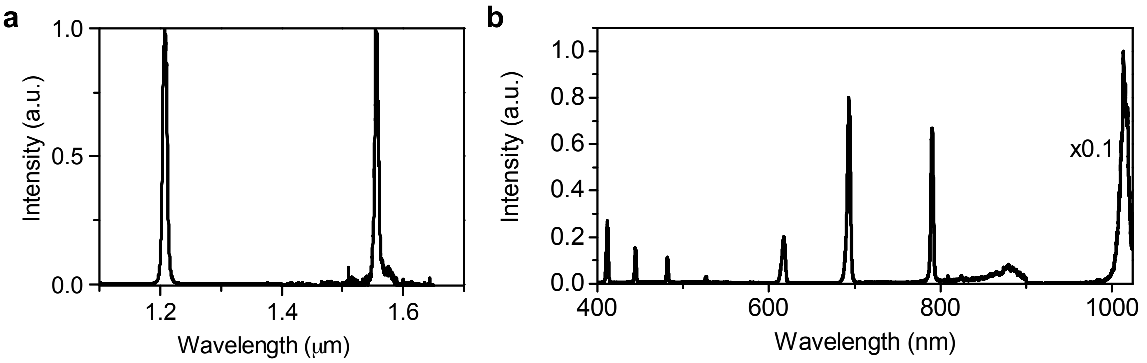

Supplementary Figure 4. Frequency mixing spectrum generated by the metasurface pumped by two pulses with ~8.5 nm bandwidth. **a** Spectra of the two pump beams, that are transmitted through two narrow-band pass filters for a better spectral distinction. Both the filters limit the pump bandwidth to ~8.5 nm. **b** Measured nonlinear frequency mixing spectrum generated by the metasurface pumped by two pulses with ~8.5 nm bandwidth.

SHG λ_2 (nm)	SHG λ_1 (nm)	Theory λ_{SWM} (nm)	Experiment λ_{SWM} (nm)
616.83	800.2	592.14	591.6
617.45	790.6	581.4	580.1
617.4	779.3	569.29	569.3
605.8	779.44	574.5	574.4
624.36	778.73	565.78	565.6

Supplementary Table 2. Verification of six-wave mixing (SWM) process by spectrally tuning the pump beams. The experimentally measured SWM wavelengths compared with calculated ones. The fundamental pump wavelengths are calculated ($\lambda_{pump} = 2 * \lambda_{SHG}$) using the experimentally measured SHG and the SWM wavelengths are derived based on $\omega_{SWM} = 4\omega_1 - \omega_2$. The experiments were performed with the two narrow bandwidth pass filters so the wavelengths of the SHG and SWM can be identified more accurately.

Note that the nonlinear processes can be attributed to cascaded processes (for example, the third-harmonic could be generated by the sum-frequency between the second-harmonic signal and the fundamental pump instead of direct third order generation). However, it is challenging to separate the direct and cascaded processes in subwavelength resonant cavities. These results are outside of the scope of our discussion in this paper and are still under investigation.

Supplementary Note 7

Frequency mixing on an unpatterned sample as well as on other metamixer samples consisting of GaAs nanocylinders with different diameters

To confirm the resonantly enhanced frequency mixing processes, we also measured several other samples. Supplementary Figure 5a shows the reflectance spectra of three samples with different GaAs nanocylinder diameters of d = 340, 420, and 620 nm. The red and blue dashed lines indicate the central wavelengths of the two pump beams. For the metamixer comprising 620 nm diameter resonators, the MD and ED resonances are at a wavelength longer than 1600 nm and the pumps can only excite higher order resonances. For another metamixer consisting of resonators with a smaller diameter of 340 nm, the MD and ED resonances occur at wavelengths of ~1250 and ~1100 nm, respectively. The spectra of the nonlinear signal generated by these metamixers are compared in Supplementary Figure 5b. As expected, the signals generated by the 420 nm diameter metamixer are much larger than the other two metamixers for most of the nonlinear processes, except the photoluminescence signal. In addition, we measured the mixing signal using an unpatterned sample as shown in Supplementary Figure 5c. Much lower conversion efficiencies were observed especially considering that we applied ~3 times higher

pump power on the unpatterned sample. The only nonlinear processes can be identified from the unpatterned sample are the two-photon induced photoluminescence at 870 nm and two of the four-wave mixing signals at 440 nm and 480 nm, respectively.

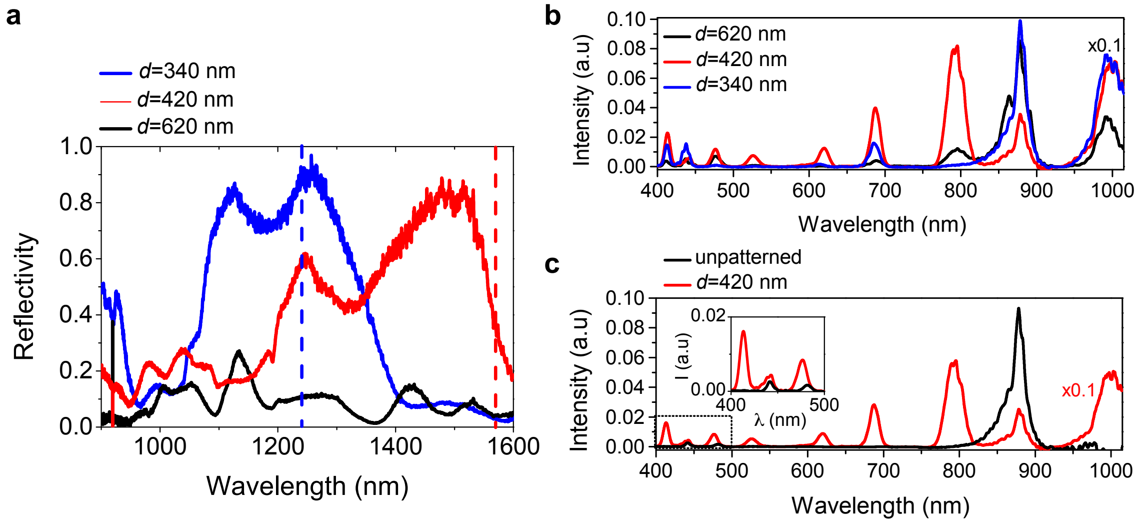

Supplementary Figure 5. Measurements of the frequency mixing spectra from different samples. **a** Reflectance spectra of three metamixers with different diameters of the nanocylinders of *d*=340 nm (blue), *d*=420 nm (red) and *d*=620 nm (black). Dashed lines indicate the central wavelengths of the two pump beams. **b** The generated nonlinear mixing spectra from the three different metamixers. **c** Nonlinear mixing spectrum generated from unpatterned region (black curve) compared with the spectrum generated by the metamixer with *d*=420 nm (red curve). Insert is the zoom-in of generated third-harmonic and four-wave mixing signals in the spectral range from 400 to 500 nm.

Supplementary Note 8

Time-dependent measurements

To measure the temporal dynamics of frequency mixing processes, we varied the delay between the two pump pulses while collecting the nonlinear mixing spectra. The 2D contour image in

Figure 3a of the main text shows the transient nonlinear signal between -300 fs and +300 fs. Note that negative delay indicates that the second ($\lambda_2 \sim 1240$ nm) pump beam arrived at the metamixer ahead of the first ($\lambda_1 \sim 1570$ nm) pump beam and positive delay indicates the first pump beam arriving first. First, as stated in the main text, the mixing signals that involve both pump beams were generated only when the two pump pulses overlap temporally. More interestingly, these signals show a delay dependent spectral shift. For example, the sum-frequency generation peak blue shifts as the delay moves from negative to positive. This spectral shift is likely caused by the chirp of the pump beams, as illustrated in Supplementary Figure 6a[10,11]. When the time delay is negative – only longer wavelengths play a role. To verify this, the pump pulses were characterized by a frequency-resolved optical gating (FROG) setup (Mesa-Photonics, LLC). The measured SHG FROG trace (Supplementary Figure 6b) and reconstructed time-dependent intensity and phase (Supplementary Figure 6c) of ω_2 pulse indeed show the presence of the chirp.

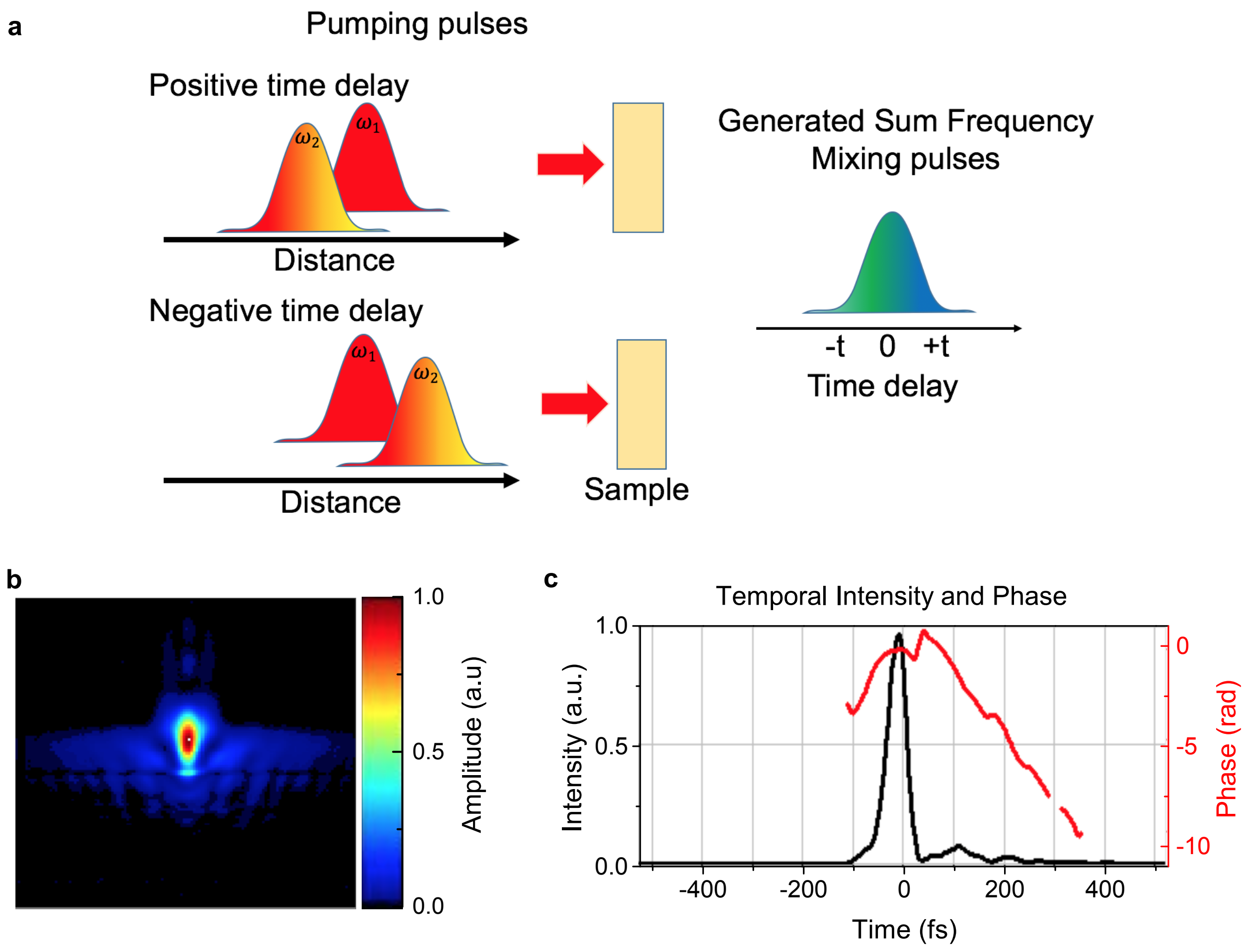

Supplementary Figure 6. Characterization of the pumping pulse in the time-domain. **a** Illustration of sum-frequency generation when the second pump pulse has a negative chirp. The sum frequency is generated only by the overlapping parts of the pulses: in the case of positive delay, shorter wavelengths components of the ω_2 contribute to the SFG signal; and in the case of negative delay – longer wavelengths components of the ω_2 contribute to the SFG signal. This can lead to the experimentally observed time-dependent spectral shift. **b** Experimentally measured trace for ω_2 pulse by second-harmonic based frequency-resolved optical gating (SHG FROG) method. **c** Time-dependent intensity (black curve) and phase (red curve) obtained from the data in (**b**), verifying the presence of chirp in the laser pulse.

Supplementary References:

1 M. W. Klein, C. E., M. Wegener, S. Linden. Second-Harmonic Generation from Magnetic Metamaterials. *Science* **313**, 502-504 (2006).

2 Celebrano, M. *et al.* Mode matching in multiresonant plasmonic nanoantennas for enhanced second harmonic generation. *Nat Nano* **10**, 412-417 (2015).

3 Liu, S. *et al.* Resonantly Enhanced Second-Harmonic Generation Using III–V Semiconductor All-Dielectric Metasurfaces. *Nano Letters* **16**, 5426-5432 (2016).

4 Camacho-Morales, R. *et al.* Nonlinear Generation of Vector Beams From AlGaAs Nanoantennas. *Nano Letters* **16**, 7191-7197 (2016).

5 Klein, M. W., Wegener, M., Feth, N. & Linden, S. Experiments on second- and third-harmonic generation from magnetic metamaterials. *Opt. Express* **15**, 5238-5247 (2007).

6 Shcherbakov, M. R. *et al.* Enhanced Third-Harmonic Generation in Silicon Nanoparticles Driven by Magnetic Response. *Nano Letters* **14**, 6488-6492 (2014).

7 Palomba, S. *et al.* Optical negative refraction by four-wave mixing in thin metallic nanostructures. *Nat Mater* **11**, 34-38 (2012).

8 Grinblat, G., Li, Y., Nielsen, M. P., Oulton, R. F. & Maier, S. A. Degenerate Four-Wave Mixing in a Multiresonant Germanium Nanodisk. *ACS Photonics* **4**, 2144-2149 (2017).

9 Sartorello, G. *et al.* Ultrafast Optical Modulation of Second- and Third-Harmonic Generation from Cut-Disk-Based Metasurfaces. *ACS Photonics* **3**, 1517-1522 (2016).

10 DeLong, K. W., Trebino, R., Hunter, J. & White, W. E. Frequency-resolved optical gating with the use of second-harmonic generation. *Journal of the Optical Society of America B* **11**, 2206-2215 (1994).

11 Trebino, R. *et al.* Measuring ultrashort laser pulses in the time-frequency domain using frequency-resolved optical gating. *Review of Scientific Instruments* **68**, 3277-3295 (1997).
